# Crystal structures of 3,3′-bis­(hy­droxy­dimethylsilan­yl)azo­benzene and 4,4′-bis­(hy­droxy­dimethyl­silane)azo­benzene

**DOI:** 10.1107/S2056989016016297

**Published:** 2016-10-18

**Authors:** Jan Strüben, Jonas Hoffmann, David Presa-Soto, Christian Näther, Anne Staubitz

**Affiliations:** a Otto-Diels-Institut für Organische Chemie, Christian-Albrechts-Universität Kiel, Otto-Hahn-Platz 4, 24118 Kiel, Germany; b Department of Organic and Inorganic Chemistry IUQOEM, University of Oviedo, Julián Claveria, 33006 Oviedo, Spain; c Institut für Anorganische Chemie, Christian-Albrechts-Universität Kiel, Max-Eyth-Strasse 2, 24118 Kiel, Germany; d Institute for Organic and Analytical Chemistry, University of Bremen, Leobener Strasse NW2 C, 28359 Bremen, Germany; e MAPEX Center for Materials and Processes University of Bremen, Bibliothekstrasse 1, 28359 Bremen, Germany

**Keywords:** crystal structure, azo­benzene, O—H⋯O hydrogen bonding

## Abstract

The title compounds {systematic names (*E*)-[diazene-1,2-diylbis(3,1-phenyl­ene)]bis­(di­methyl­silanol) and (*E*)-[diazene-1,2-diylbis(4,1-phenyl­ene)]bis­(di­methyl­silanol)}, both of the sum formula C_16_H_22_N_2_O_2_Si_2_, were obtained by transmetallation of the respective bis-stannylated azo­benzenes with di­chloro­dimethyl­silane and esterification followed by hydrolysis. The asymmetric unit of 3,3′-diazenediylbis[dimeth­yl(phen­yl)silanol] (with the silanol functional group in a *meta* position) consists of two mol­ecules, of which one occupies a general position, whereas the second is located on a centre of inversion. In 4,4′-diazenediylbis[dimeth­yl(phen­yl)silanol] (with the silanol functional group in a *para* position) likewise two mol­ecules are present in the asymmetric unit, but in this case both occupy general positions. Differences between all mol­ecules can be found in the torsions about the N=N bond, as well as in the dihedral angles between the benzene rings. In both structures, inter­molecular O—H⋯O hydrogen bonding is observed, leading to the formation of layers parallel to (01-1) for (I) and to chains parallel to the *a* axis for (II).

## Abstract 2

In each of the crystal structures of the two title compounds, two mol­ecules are found in the asymmetric unit. Individual mol­ecules are linked by inter­molecular O—H⋯O hydrogen bonding and show significant differences in the torsions about the N=N bond and the dihedral angle between the benzene rings.

## Chemical context   

Azo­benzenes have been widely investigated as photoswitches due to their photochemically induced *trans*/*cis*-isomerization. Furthermore, they are common motifs in dyes due to their high thermal and photochemical stability (Yesodha *et al.*, 2004 ▸; Lagrasta *et al.*, 1997 ▸). Their application as mol­ecular switches is sometimes limited by their synthetical accessability. For *ortho*, *meta* and *para*-substituted azo­benzenes, a novel functionalization has been presented recently (Strüben *et al.*, 2014 ▸, 2015 ▸). This opens access to new synthetic pathways and hence new dyes and materials, for example light-responsive polymers (Yu *et al.*, 2003 ▸; Kizilkan *et al.*, 2016 ▸).
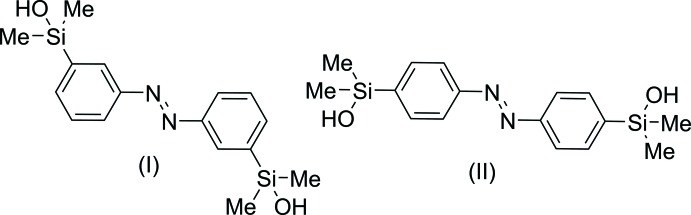


In the above context, we report here on the synthesis and crystal structures of two regioisomers with composition C_16_H_22_N_2_O_2_Si_2_, obtained by transmetalation of the respective bis-stannylated azo­benzenes.

## Structural commentary   

**Figure 1:**
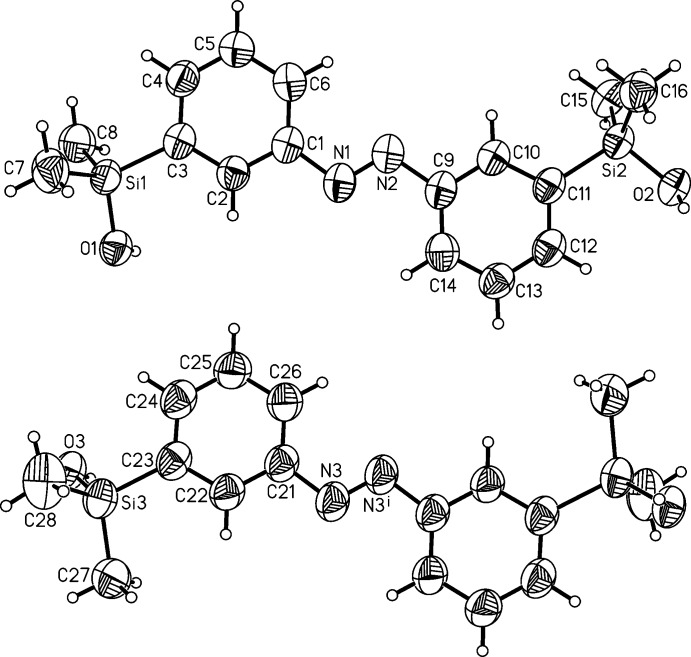
The mol­ecular structures of the two crystallographically independent mol­ecules in the crystal structure of isomer (I) (*a* top and *b* bottom) with labelling and displacement ellipsoids drawn at the 50% probability level. Symmetry code for the generation of equivalent atoms: −*x* + 2, −*y* + 2, −*z* + 2.

The crystal structures of the *meta-* (I) and *para-*substituted (II) azo­benzenes each comprise two crystallographically independent mol­ecules [(I*a*) and centrosymmetric (I*b*), and (II*a*) and (II*b*), respectively; Figs. 1 ▸ and 2 ▸). With respect to the central N=N bond, the azogroups are *trans* configured. The N=N bond lengths in all mol­ecules [1.256 (3) Å for (I*a*), 1.250 (5) Å for (I*b*), 1.246 (2) for (II*a*) and 1.248 (2) Å for (II*b*)] are comparable and agree well with values retrieved from the literature (Groom *et al.*, 2016 ▸). Differences between the independent mol­ecules are found, *e.g.* in the C—N=N—C torsion angles which amount to −178.6 (2)° in (I*a*) and due to symmetry restrictions to 0° in (I*b*). For mol­ecules of isomer (II) values of −177.93 (14)° (II*a*) and 178.47 (14)° (II*b*) are observed. In mol­ecule (I*b*), the benzene rings are coplanar (dihedral angle = 0°), whereas in (I*a*) they are rotated by 11.87 (14)°. In isomer (II), values of 27.40 (8)° (II*a*) and 17.28 (9)° (II*b*) are found for the two mol­ecules.

## Supra­molecular features   

**Figure 3:**
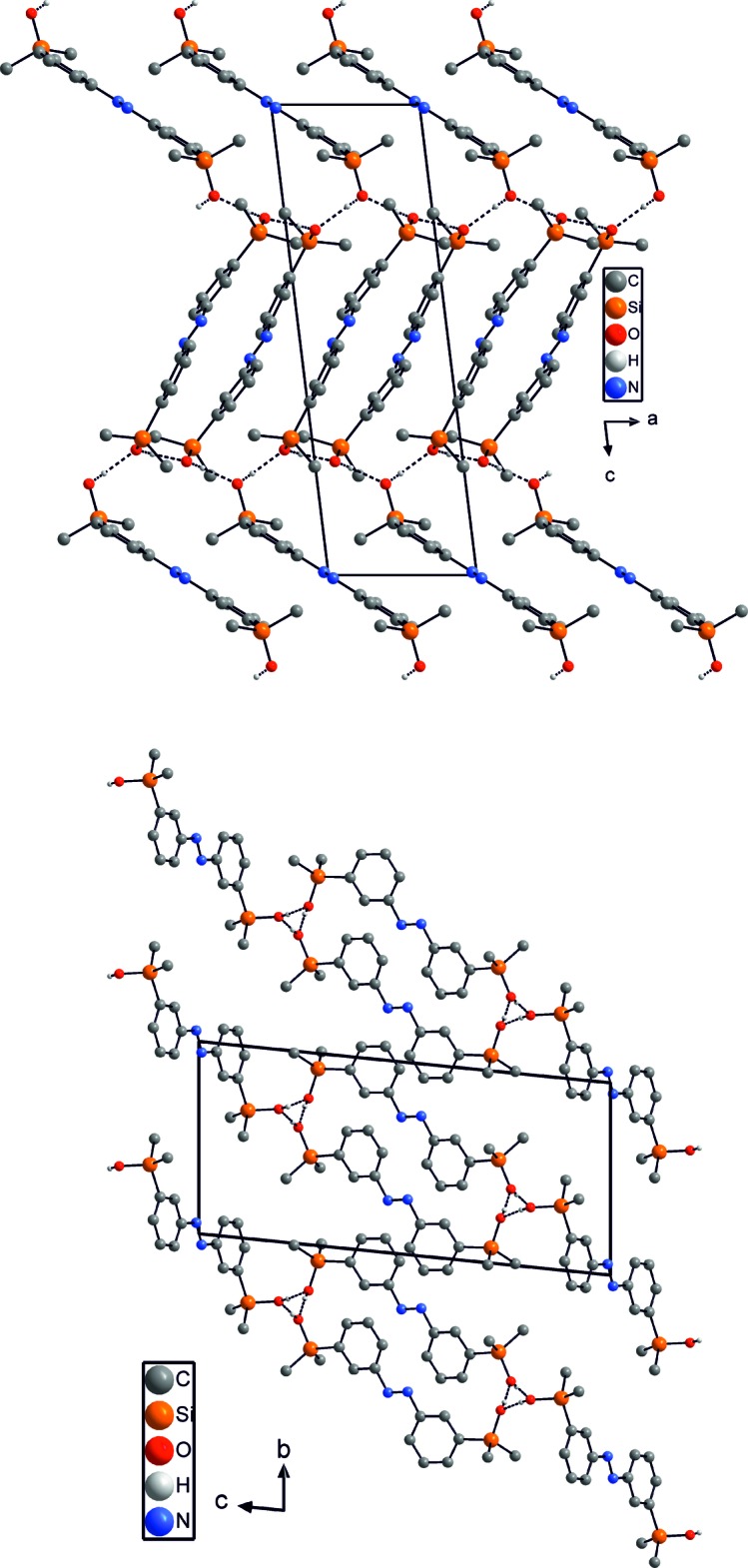
Crystal structure of isomer (I) in a view along the crystallographic *b* axis (top) and along the *a* axis (bottom). Inter­molecular O—H⋯O hydrogen bonding is shown as dashed lines and C—H hydrogen atoms have been omitted for clarity.

**Figure 4:**
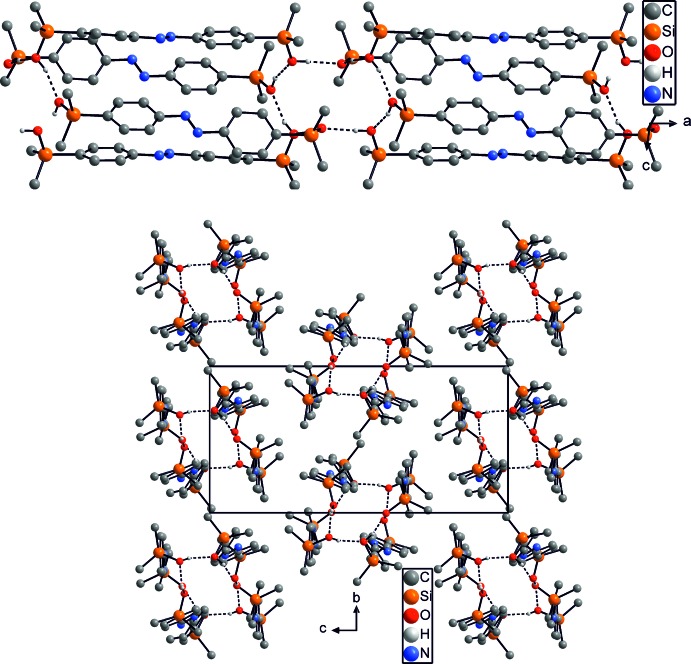
Crystal structure of isomer (II) showing a view of the hydrogen-bonded chains (top) and along the crystallographic *b* axis (bottom). Inter­molecular O—H⋯O hydrogen bonding is shown as dashed lines and C—H hydrogen atoms have been omitted for clarity.

In the crystal structure of isomer (I), neighboring mol­ecules are linked by inter­molecular O—H⋯O hydrogen bonding between the silyl­hydroxyl hydrogen atoms of the first independent mol­ecules, forming chains that elongate in the *a-*axis direction (Fig. 3 ▸ top). These chains are further linked *via* O—H⋯O hydrogen bonds to the second crystallographically independent mol­ecules, forming layers that are parallel to (01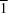) (Fig. 3 ▸, bottom, Table 1 ▸). The O—H⋯O angles and O⋯O contacts indicate that these are rather strong hydrogen bonds (Table 1 ▸). Between the layers, slipped π–π inter­actions [centroid-to-centroid distances 3.767 (2) and 3.811 (2) Å] are present, consolidating the crystal packing. In isomer (II), the mol­ecules are likewise linked by inter­molecular O—H⋯O hydrogen bonding into tetra­meric units, which are further linked into chains that elongate in the *a-*axis direction (Fig. 4 ▸, top, Table 2 ▸). By this arrangement, 16-membered cyclic hydrogen-bonded motifs are formed that consist of eight alternating hy­droxy­silyl groups and that can be described as 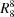
(16) according to the graph-set notation (Etter *et al.*, 1990 ▸; Bernstein *et al.*, 1995 ▸). As in isomer (I), the values of the O—H⋯O angles and O⋯O distances indicate rather strong hydrogen bonding (Table 2 ▸). These tetra­meric chains are packed along the *a* axis in a pseudo-hexa­gonal arrangement (Fig. 4 ▸, bottom).

## Database Survey   

Hundreds of azobenze-based structures are found in the Cambridge Structural Database (Groom *et al.*, 2016 ▸) but compounds with silanol groups are unknown (*ConQuest* Version 1.18, CSD Version 5.37). There are also no compounds reported with silyl groups in a *meta* or a *para* position but some compounds have been deposited in which both benzene rings are substituted in the *ortho* position by, *e.g.*, tri­methyl­silyl, fluoro-di­methyl­silyl, di­fluoro-methyl­silyl or tri­fluoro­silyl groups (Kano *et al.*, 2001 ▸). It is noted that two structures are reported in which two azobenenzene mol­ecules are bridged by Si—O—Si groups in the *ortho* position (Kano *et al.*, 2003 ▸; Yamamura *et al.*, 2009 ▸).

## Synthesis and crystallization   

The syntheses of 3,3′-bis­(tri­methyl­stann­yl)azo­benzene and 4,4′-bis­(tri­methyl­stann­yl)azo­benzene were described in the literature (Strüben *et al.*, 2014 ▸). For further details of the transmetallation, see: Strüben *et al.* (2015 ▸). Di­methyldi­chloro­silane (99%) was purchased from ABCR Inc., degassed and distilled from calcium hydride. Methyl lithium (1.6 *M* in diethyl ether) was purchased from Acros Organics, monopotassium phosphate (99.7%) was purchased from Sigma–Aldrich, sodium methoxide (99%) from TCI Inc. and used without further purification. THF was purchased from Merck–Polaro and was dried and degassed with a PS-MD-5 by Innovation Technology. Methanol as obtained from BCD was distilled from sodium and was stored over mol­ecular sieves (3 Å).

**3,3′-Bis(Hy­droxy­dimethyl­silane)azo­benzene**

3,3′-Bis(tri­methyl­stann­yl)azo­benzene (3.80 g, 7.48 mmol) was dissolved in dry THF (100 ml). Then, at 195 K, methyl lithium (12.0 ml, 19.0 mmol, 1.6 *M* solution in diethyl ether) in THF (18.0 ml) was added and the mixture was stirred for 10 min at 195 K. Then the reaction was quenched with di­chloro­dimethyl­silane (30.0 ml, 32.1 g, 249 mmol) and the reaction mixture allowed to warm to 298 K in a cooling bath. Subsequently the solvent and the excess of di­chloro­dimethyl­silane were evaporated in inert conditions under reduced pressure. The residual orange solid was dissolved in diethyl ether (25 ml) and added dropwise over the course of 15 min to a solution of sodium methoxide (4.00 g, 74.0 mmol) in methanol (50 ml). Both of the latter steps were performed under inert conditions. To this mixture, a solution of sodium hydroxide (17.5 g, 438 mmol) in methanol (105 ml) and water (10.0 ml) was added. The resulting solution was stirred for 15 minutes and then a further portion of sodium hydroxide (17.5 g, 438 mmol) in water (105 ml) was added. The reaction mixture was stirred for 1 h. This mixture was finally poured into a vigorously stirred solution of monopotassium phosphate (155 g, 1.14 mol) in water (200 ml). The orange precipitate was filtered and purified by three recrystallization cycles from diethyl ether/*n*–hexane (*v/v* 1:1). The final product was isolated as an orange solid in a yield of 500 mg (20%). Crystals were obtained by dissolving the product in chlroroform, adding a layer of *n*-hexane and allowing the *n*-hexane to diffuse into the chloroform, leading to crystal formation at the phase boundary.

**^1^H NMR** (500 MHz, CDCl_3_): δ = 8.14 (at, ^4^ *J* = 4.6 Hz, 2 H, H–2), 7.92 (adt, ^3^ *J* = 7.9 Hz, 4*J* = 4.6 Hz, 2 H, *H*–4), 7.70 (adt, ^3^ *J* = 7.9 Hz, 4*J* = 4.6 Hz, 2 H, *H*–6), 7.53 (atd, ^3^ *J* = 7.9 Hz, 2 H, *H*–5), 2.5 (*s*, 2 H, O*H*), 0.46 (*s*, 18 H, *H*–7) p.p.m.

**^13^C NMR** (126 MHz, CDCl_3_): δ = 152.0 (*C*–3), 140.5 (*C*–1), 135.7 (*C*–6), 128.8 (*C*–5), 128.0 (*C*–2), 123.4 (*C*–4), 0.2 (*C*–8) p.p.m.

**^29^Si NMR** (187 MHz, CDCl_3_): δ = 7.61 p.p.m.

**IR** (ATR): ν = 3189 (*m*), 2955 (*w*), 1398 (*m*), 1251 (*m*), 1111 (*w*), 1068 (*m*), 897 (*s*), 863 (*s*), 820 (*s*), 799 (*s*), 764 (*s*), 691 (*s*), 645 (*m*), 534 (*m*) cm^−1^.

**HRMS** (EI–sector) *m/z*: [*M*]^+^ calculated for [C_16_H_22_N_2_O_2_Si_2_]^+^ 330.1220, found 330.1222.

**M.p.:** *T* = 374 K.

**4,4′-Bis(hy­droxy­dimethyl­silane)azo­benzene**

4,4′-Bis(tri­methyl­stann­yl)azo­benzene (3.80 g, 7.48 mmol) was dissolved in dry THF (100 ml). A solution of methyl lithium (12.0 ml, 19.0 mmol, 1.6 *M* solution in diethyl ether) in THF (18.0 ml) was added at 195 K. The orange solution turned dark and was stirred for 10 min. Then di­chloro­dimeth­yl­silane (30.0 ml, 32.1 g, 249 mmol) was added to quench the reaction and the reaction mixture allowed to warm to 298 K in a cooling bath. Then the solvent and the excess of di­chloro­dimethyl­silane were evaporated in inert conditions under reduced pressure. The residual orange solid was dissolved in diethyl ether (25 ml) and added dropwise over the course of 15 min to a solution of sodium methoxide (4.00 g, 74.0 mmol) in methanol (50 ml). Both of the latter steps were performed under inert conditions. To this mixture, a solution of sodium hydroxide (17.5 g, 435 mmol) in methanol (105 ml) and water (10 ml) was added. The resulting mixture was stirred 15 minutes and then a further portion of sodium hydroxide (17.5 g) in water (105 ml) was added. The reaction mixture was stirred for 1 h. This mixture was then poured into a vigorously stirred solution of monopotassium phosphate (155 g, 1.14 mol) in water (200 ml). The orange precipitate was filtered and purified by three recrystallization cycles from diethyl ether/*n*-hexane (*v/v*, 1:1). The product was isolated as a bright-orange solid in a yield of 864 mg (35%). Crystals were obtained by dissolving the product in chlroroform, adding a layer of *n*-hexane and allowing the *n*-hexane to diffuse into the chloroform, leading to crystal formation at the phase boundary.

**^1^H NMR** (500 MHz, CDCl_3_): δ = 7.92 (*m*, 4 H, *H*–3, 3′), 7.75 (*m*, 4 H, *H*–2, 2′), 1.99 (*s*, 2H, O*H*), 0.46 (*s*, 12 H, *H*–5) p.p.m.

**^13^C NMR** (126 MHz, CDCl_3_): δ = 153.3 (*C*–4), 142.8 (*C*–1), 133.9 (*C*–2,2′), 122.1 (*C*–3,3′), 0.2 (*C*–5) p.p.m.

**^29^Si NMR** (187 MHz, CDCl_3_): δ = 7.77 p.p.m.

**IR** (ATR): ν = 3141 (*m*), 2956 (*w*), 1385 (*m*), 1251 (*m*), 1106 (*w*), 859 (*s*), 833 (*s*), 815 (*s*), 776 (*s*), 667 (*s*), 553 (*s*), 529 (*m*), 491 (*m*) cm^−1^.

**HRMS** (EI–sector) *m/z*: [*M*]^+^ calculated for [C_16_H_22_N_2_O_2_Si_2_]^+^ 330.1220, found 330.1221.

**M.p.:** *T* **=** 414 K.

## Refinement   

Crystal data, data collection and structure refinement details are summarized in Table 3 ▸. All C- and O-bound H atoms were located in difference maps but were positioned with idealized geometry (methyl and hydroxyl H atoms allowed to rotate but not to tip) and refined with *U*_iso_(H) = 1.2*U*_eq_(C) (1.5 for methyl and hydroxyl H atoms) using a riding model.

## Supplementary Material

Crystal structure: contains datablock(s) I, II, global. DOI: 10.1107/S2056989016016297/wm5327sup1.cif

Structure factors: contains datablock(s) I. DOI: 10.1107/S2056989016016297/wm5327Isup2.hkl

Structure factors: contains datablock(s) II. DOI: 10.1107/S2056989016016297/wm5327IIsup3.hkl

Supporting information file. DOI: 10.1107/S2056989016016297/wm5327Isup4.cml

Supporting information file. DOI: 10.1107/S2056989016016297/wm5327IIsup5.cml

CCDC references: 1509708, 1509707

Additional supporting information:  crystallographic information; 3D view; checkCIF report

## Figures and Tables

**Figure 2:**
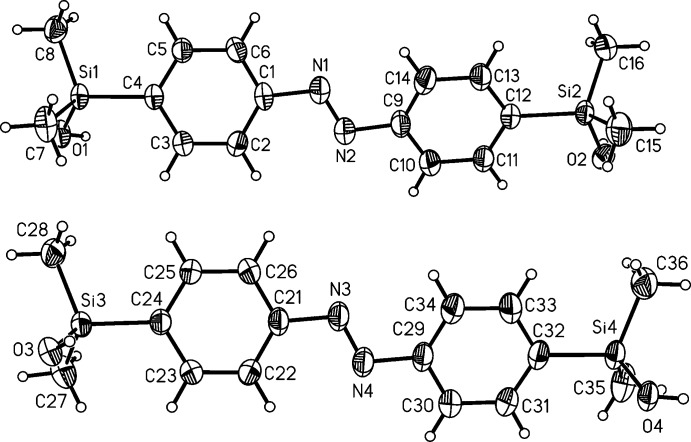
The mol­ecular structure of the two crystallographically independent mol­ecules in the crystal structure of isomer (II) (*a* top and *b* bottom) with labelling and displacement ellipsoids drawn at the 50% probability level.

**Table 1:** Hydrogen-bond geometry (Å, °) for (I)

*D*—H⋯*A*	*D*—H	H⋯*A*	*D*⋯*A*	*D*—H⋯*A*
O1—H1*O*⋯O2^i^	0.84	1.87	2.708 (3)	173
O2—H2*O*⋯O3	0.84	1.86	2.701 (3)	177
O3—H3*O*⋯O1^ii^	0.84	1.86	2.696 (3)	175

Symmetry codes: (i) 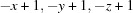; (ii) 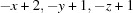.

**Table 2:** Hydrogen-bond geometry (Å, °) for (II)

*D*—H⋯*A*	*D*—H	H⋯*A*	*D*⋯*A*	*D*—H⋯*A*
O1—H1*O*⋯O3^i^	0.84	1.86	2.6686 (15)	161
O2—H2*O*⋯O4^i^	0.84	1.92	2.7297 (16)	160
O3—H3*O*⋯O2^ii^	0.84	1.90	2.7010 (15)	160
O4—H4*O*⋯O1^iii^	0.84	1.87	2.7063 (14)	175

Symmetry codes: (i) 
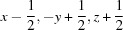
; (ii) 
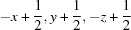
; (iii) 
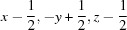
.

**Table 3:** Experimental details

	(I)	(II)
Crystal data		
Chemical formula	C_16_H_22_N_2_O_2_Si_2_	C_16_H_22_N_2_O_2_Si_2_
*M*_r_	330.53	330.53
Crystal system, space group	Triclinic, *P* 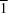	Monoclinic, *P*2_1_/*n*
Temperature (K)	200	200
*a*, *b*, *c* (Å)	6.6731 (4), 9.8806 (6), 21.4108 (10)	17.8705 (4), 10.0016 (3), 20.5323 (5)
α, β, γ (°)	83.992 (4), 82.810 (4), 87.508 (5)	90, 97.013 (2), 90
*V* (Å^3^)	1392.25 (14)	3642.36 (16)
*Z*	3	8
Radiation type	Mo *K*α	Mo *K*α
μ (mm^−1^)	0.20	0.20
Crystal size (mm)	0.30 × 0.20 × 0.10	0.15 × 0.15 × 0.10

Data collection		
Diffractometer	Stoe IPDS2	Stoe IPDS2
Absorption correction	Numerical (*X-RED32* and *X-SHAPE*; Stoe, 2008 ▸)	–
*T*_min_, *T*_max_	0.850, 0.974	–
No. of measured, independent and observed [*I* > 2σ(*I*)] reflections	11236, 4845, 3542	30514, 7877, 6720
*R*_int_	0.040	0.026
(sin θ/λ)_max_ (Å^−1^)	0.595	0.639

Refinement		
*R*[*F* ^2^ > 2σ(*F* ^2^)], *wR*(*F* ^2^), *S*	0.050, 0.126, 1.03	0.037, 0.096, 1.04
No. of reflections	4845	7877
No. of parameters	308	409
H-atom treatment	H-atom parameters constrained	H-atom parameters constrained
Δρ_max_, Δρ_min_ (e Å^−3^)	0.22, −0.26	0.32, −0.20

Computer programs: *X-AREA* (Stoe, 2008 ▸), *SHELXS97* (Sheldrick, 2008 ▸), *SHELXL2014* (Sheldrick, 2015 ▸), *XP* in *SHELXTL* (Sheldrick, 2008 ▸), *DIAMOND* (Brandenburg, 1999 ▸) and *publCIF* (Westrip, 2010 ▸).

## supplementary crystallographic information

### (I) (E)-[Diazene-1,2-diylbis(3,1-phenylene)]bis(dimethylsilanol) Crystal data

**Table:**

C_16_H_22_N_2_O_2_Si_2_	*Z* = 3
*M**_r_* = 330.53	*F*(000) = 528
Triclinic, *P*1	*D*_x_ = 1.183 Mg m^−^^3^
*a* = 6.6731 (4) Å	Mo *K*α radiation, λ = 0.71073 Å
*b* = 9.8806 (6) Å	Cell parameters from 11236 reflections
*c* = 21.4108 (10) Å	θ = 1.9–25.0°
α = 83.992 (4)°	µ = 0.20 mm^−^^1^
β = 82.810 (4)°	*T* = 200 K
γ = 87.508 (5)°	Block, yellow-orange
*V* = 1392.25 (14) Å^3^	0.30 × 0.20 × 0.10 mm

### (I) (E)-[Diazene-1,2-diylbis(3,1-phenylene)]bis(dimethylsilanol) Data collection

**Table:**

Stoe IPDS-2 diffractometer	3542 reflections with *I* > 2σ(*I*)
ω scan	*R*_int_ = 0.040
Absorption correction: numerical (X-RED32 and X-SHAPE; Stoe, 2008)	θ_max_ = 25.0°, θ_min_ = 1.9°
*T*_min_ = 0.850, *T*_max_ = 0.974	*h* = −7→7
11236 measured reflections	*k* = −9→11
4845 independent reflections	*l* = −25→25

### (I) (E)-[Diazene-1,2-diylbis(3,1-phenylene)]bis(dimethylsilanol) Refinement

**Table:**

Refinement on *F*^2^	Hydrogen site location: inferred from neighbouring sites
Least-squares matrix: full	H-atom parameters constrained
*R*[*F*^2^ > 2σ(*F*^2^)] = 0.050	*w* = 1/[σ^2^(*F*_o_^2^) + (0.0567*P*)^2^ + 0.3154*P*] where *P* = (*F*_o_^2^ + 2*F*_c_^2^)/3
*wR*(*F*^2^) = 0.126	(Δ/σ)_max_ = 0.001
*S* = 1.03	Δρ_max_ = 0.22 e Å^−^^3^
4845 reflections	Δρ_min_ = −0.26 e Å^−^^3^
308 parameters	Extinction correction: SHELXL2014 (Sheldrick, 2015), Fc^*^=kFc[1+0.001xFc^2^λ^3^/sin(2θ)]^-1/4^
0 restraints	Extinction coefficient: 0.029 (4)

### (I) (E)-[Diazene-1,2-diylbis(3,1-phenylene)]bis(dimethylsilanol) Special details

**Table:**

Geometry. All esds (except the esd in the dihedral angle between two l.s. planes) are estimated using the full covariance matrix. The cell esds are taken into account individually in the estimation of esds in distances, angles and torsion angles; correlations between esds in cell parameters are only used when they are defined by crystal symmetry. An approximate (isotropic) treatment of cell esds is used for estimating esds involving l.s. planes.

### (I) (E)-[Diazene-1,2-diylbis(3,1-phenylene)]bis(dimethylsilanol) Fractional atomic coordinates and isotropic or equivalent isotropic displacement parameters (Å^2^)

**Table:**

	*x*	*y*	*z*	*U*_iso_*/*U*_eq_	
C1	0.8173 (4)	0.1655 (3)	0.46148 (13)	0.0589 (7)	
C2	0.9324 (4)	0.1796 (3)	0.40274 (12)	0.0588 (7)	
H2	0.9793	0.2669	0.3858	0.071*	
C3	0.9809 (4)	0.0679 (3)	0.36788 (12)	0.0590 (7)	
C4	0.9071 (5)	−0.0579 (3)	0.39497 (13)	0.0657 (7)	
H4	0.9367	−0.1356	0.3724	0.079*	
C5	0.7927 (5)	−0.0728 (3)	0.45353 (14)	0.0679 (8)	
H5	0.7448	−0.1597	0.4705	0.082*	
C6	0.7477 (4)	0.0383 (3)	0.48736 (13)	0.0641 (7)	
H6	0.6702	0.0283	0.5278	0.077*	
Si1	1.14136 (12)	0.07944 (8)	0.28970 (4)	0.0599 (2)	
O1	1.1934 (3)	0.23933 (19)	0.26646 (9)	0.0620 (5)	
H1O	1.0878	0.2822	0.2574	0.093*	
C7	1.3851 (5)	−0.0109 (4)	0.29776 (17)	0.0863 (10)	
H7A	1.4698	−0.0033	0.2568	0.129*	
H7B	1.3620	−0.1071	0.3119	0.129*	
H7C	1.4535	0.0297	0.3289	0.129*	
C8	1.0036 (6)	0.0100 (4)	0.23111 (15)	0.0858 (10)	
H8A	0.8789	0.0642	0.2265	0.129*	
H8B	0.9701	−0.0847	0.2455	0.129*	
H8C	1.0886	0.0135	0.1902	0.129*	
N1	0.7784 (4)	0.2872 (3)	0.49246 (11)	0.0636 (6)	
N2	0.6495 (4)	0.2720 (3)	0.54049 (11)	0.0660 (6)	
C9	0.6026 (4)	0.3926 (3)	0.57206 (13)	0.0610 (7)	
C10	0.4536 (4)	0.3763 (3)	0.62337 (12)	0.0602 (7)	
H10	0.3961	0.2895	0.6347	0.072*	
C11	0.3852 (4)	0.4840 (3)	0.65906 (12)	0.0576 (7)	
C12	0.4745 (4)	0.6092 (3)	0.63984 (13)	0.0624 (7)	
H12	0.4325	0.6850	0.6626	0.075*	
C13	0.6230 (5)	0.6259 (3)	0.58829 (13)	0.0646 (7)	
H13	0.6802	0.7125	0.5763	0.077*	
C14	0.6878 (4)	0.5178 (3)	0.55437 (13)	0.0642 (7)	
H14	0.7899	0.5293	0.5193	0.077*	
Si2	0.18738 (12)	0.45838 (8)	0.72857 (4)	0.0575 (2)	
O2	0.1366 (3)	0.60480 (19)	0.75838 (9)	0.0611 (5)	
H2O	0.2396	0.6300	0.7723	0.092*	
C15	−0.0549 (5)	0.4071 (3)	0.70559 (16)	0.0727 (8)	
H15A	−0.1505	0.3862	0.7437	0.109*	
H15B	−0.0314	0.3263	0.6824	0.109*	
H15C	−0.1109	0.4818	0.6785	0.109*	
C16	0.2840 (5)	0.3318 (3)	0.78818 (14)	0.0736 (8)	
H16A	0.4026	0.3672	0.8031	0.110*	
H16B	0.3222	0.2470	0.7691	0.110*	
H16C	0.1786	0.3140	0.8240	0.110*	
C21	0.8037 (5)	0.9557 (3)	0.95728 (13)	0.0678 (8)	
C22	0.7417 (5)	0.8341 (3)	0.94069 (13)	0.0690 (8)	
H22	0.8111	0.7519	0.9535	0.083*	
C23	0.5797 (5)	0.8287 (3)	0.90556 (13)	0.0687 (8)	
C24	0.4812 (5)	0.9522 (4)	0.88898 (14)	0.0749 (9)	
H24	0.3698	0.9524	0.8653	0.090*	
C25	0.5405 (5)	1.0746 (4)	0.90590 (15)	0.0776 (9)	
H25	0.4691	1.1568	0.8942	0.093*	
C26	0.7043 (5)	1.0774 (4)	0.93990 (14)	0.0757 (9)	
H26	0.7473	1.1611	0.9510	0.091*	
Si3	0.49665 (14)	0.66640 (10)	0.88091 (4)	0.0702 (3)	
O3	0.4598 (3)	0.6900 (2)	0.80611 (8)	0.0677 (5)	
H3O	0.5699	0.7075	0.7837	0.102*	
C27	0.6904 (6)	0.5296 (4)	0.8920 (2)	0.0997 (12)	
H27A	0.6439	0.4448	0.8795	0.150*	
H27B	0.7132	0.5164	0.9366	0.150*	
H27C	0.8169	0.5549	0.8658	0.150*	
C28	0.2455 (6)	0.6217 (5)	0.92350 (17)	0.1031 (13)	
H28A	0.1436	0.6892	0.9099	0.155*	
H28B	0.2491	0.6210	0.9691	0.155*	
H28C	0.2108	0.5314	0.9140	0.155*	
N3	0.9716 (4)	0.9442 (3)	0.99348 (12)	0.0740 (7)	

### (I) (E)-[Diazene-1,2-diylbis(3,1-phenylene)]bis(dimethylsilanol) Atomic displacement parameters (Å^2^)

**Table:**

	*U*^11^	*U*^22^	*U*^33^	*U*^12^	*U*^13^	*U*^23^
C1	0.0606 (16)	0.0613 (17)	0.0564 (15)	0.0072 (13)	−0.0107 (12)	−0.0128 (13)
C2	0.0612 (16)	0.0597 (17)	0.0559 (15)	0.0026 (12)	−0.0085 (12)	−0.0074 (12)
C3	0.0614 (16)	0.0602 (17)	0.0571 (15)	0.0065 (13)	−0.0120 (12)	−0.0126 (13)
C4	0.0732 (18)	0.0611 (18)	0.0636 (17)	0.0021 (14)	−0.0063 (14)	−0.0146 (14)
C5	0.0716 (18)	0.0658 (19)	0.0658 (17)	−0.0038 (14)	−0.0040 (14)	−0.0085 (14)
C6	0.0618 (17)	0.073 (2)	0.0578 (16)	0.0028 (14)	−0.0060 (13)	−0.0108 (14)
Si1	0.0667 (5)	0.0561 (5)	0.0563 (4)	0.0066 (3)	−0.0036 (4)	−0.0103 (3)
O1	0.0623 (11)	0.0595 (12)	0.0640 (11)	0.0066 (9)	−0.0075 (9)	−0.0082 (9)
C7	0.085 (2)	0.081 (2)	0.084 (2)	0.0239 (18)	0.0051 (18)	0.0033 (17)
C8	0.108 (3)	0.087 (2)	0.0633 (18)	−0.015 (2)	−0.0026 (18)	−0.0195 (17)
N1	0.0657 (14)	0.0709 (16)	0.0557 (13)	0.0081 (11)	−0.0102 (11)	−0.0142 (11)
N2	0.0680 (15)	0.0708 (16)	0.0600 (14)	0.0053 (12)	−0.0050 (12)	−0.0162 (11)
C9	0.0654 (17)	0.0649 (18)	0.0548 (15)	0.0078 (13)	−0.0114 (13)	−0.0148 (13)
C10	0.0653 (17)	0.0583 (17)	0.0589 (16)	0.0036 (13)	−0.0112 (13)	−0.0119 (13)
C11	0.0627 (16)	0.0575 (17)	0.0556 (15)	0.0072 (12)	−0.0170 (12)	−0.0120 (12)
C12	0.0704 (18)	0.0606 (18)	0.0593 (16)	0.0093 (14)	−0.0186 (14)	−0.0124 (13)
C13	0.0748 (19)	0.0587 (17)	0.0621 (16)	0.0011 (14)	−0.0157 (14)	−0.0075 (13)
C14	0.0657 (17)	0.073 (2)	0.0545 (15)	0.0036 (14)	−0.0114 (13)	−0.0084 (14)
Si2	0.0626 (5)	0.0558 (5)	0.0563 (4)	0.0083 (3)	−0.0125 (3)	−0.0139 (3)
O2	0.0612 (11)	0.0605 (11)	0.0652 (11)	0.0086 (9)	−0.0150 (9)	−0.0196 (9)
C15	0.0712 (19)	0.071 (2)	0.081 (2)	0.0037 (15)	−0.0183 (16)	−0.0245 (16)
C16	0.083 (2)	0.068 (2)	0.0701 (19)	0.0099 (16)	−0.0127 (16)	−0.0087 (15)
C21	0.0696 (18)	0.080 (2)	0.0578 (16)	0.0022 (15)	−0.0166 (14)	−0.0172 (14)
C22	0.0742 (19)	0.077 (2)	0.0599 (16)	0.0046 (15)	−0.0185 (14)	−0.0172 (14)
C23	0.0694 (18)	0.088 (2)	0.0527 (15)	0.0013 (16)	−0.0142 (13)	−0.0175 (14)
C24	0.076 (2)	0.092 (2)	0.0613 (17)	0.0020 (17)	−0.0201 (15)	−0.0163 (16)
C25	0.087 (2)	0.082 (2)	0.0681 (19)	0.0087 (17)	−0.0245 (17)	−0.0164 (16)
C26	0.087 (2)	0.081 (2)	0.0642 (18)	0.0045 (17)	−0.0236 (16)	−0.0211 (16)
Si3	0.0746 (6)	0.0834 (6)	0.0560 (5)	−0.0069 (4)	−0.0152 (4)	−0.0130 (4)
O3	0.0668 (12)	0.0866 (14)	0.0530 (10)	−0.0069 (11)	−0.0113 (9)	−0.0155 (10)
C27	0.120 (3)	0.085 (3)	0.104 (3)	−0.003 (2)	−0.049 (2)	−0.011 (2)
C28	0.106 (3)	0.137 (4)	0.068 (2)	−0.031 (2)	0.002 (2)	−0.019 (2)
N3	0.0767 (16)	0.0849 (19)	0.0667 (15)	−0.0016 (14)	−0.0218 (13)	−0.0214 (13)

### (I) (E)-[Diazene-1,2-diylbis(3,1-phenylene)]bis(dimethylsilanol) Geometric parameters (Å, º)

**Table:**

C1—C2	1.386 (4)	C14—H14	0.9500
C1—C6	1.394 (4)	Si2—O2	1.6468 (18)
C1—N1	1.432 (3)	Si2—C16	1.847 (3)
C2—C3	1.400 (4)	Si2—C15	1.853 (3)
C2—H2	0.9500	O2—H2O	0.8400
C3—C4	1.398 (4)	C15—H15A	0.9800
C3—Si1	1.867 (3)	C15—H15B	0.9800
C4—C5	1.381 (4)	C15—H15C	0.9800
C4—H4	0.9500	C16—H16A	0.9800
C5—C6	1.379 (4)	C16—H16B	0.9800
C5—H5	0.9500	C16—H16C	0.9800
C6—H6	0.9500	C21—C22	1.382 (4)
Si1—O1	1.644 (2)	C21—C26	1.387 (4)
Si1—C7	1.839 (3)	C21—N3	1.434 (4)
Si1—C8	1.848 (3)	C22—C23	1.398 (4)
O1—H1O	0.8400	C22—H22	0.9500
C7—H7A	0.9800	C23—C24	1.394 (4)
C7—H7B	0.9800	C23—Si3	1.867 (3)
C7—H7C	0.9800	C24—C25	1.386 (5)
C8—H8A	0.9800	C24—H24	0.9500
C8—H8B	0.9800	C25—C26	1.389 (4)
C8—H8C	0.9800	C25—H25	0.9500
N1—N2	1.256 (3)	C26—H26	0.9500
N2—C9	1.434 (3)	Si3—O3	1.642 (2)
C9—C14	1.381 (4)	Si3—C27	1.848 (4)
C9—C10	1.387 (4)	Si3—C28	1.853 (4)
C10—C11	1.402 (4)	O3—H3O	0.8400
C10—H10	0.9500	C27—H27A	0.9800
C11—C12	1.396 (4)	C27—H27B	0.9800
C11—Si2	1.865 (3)	C27—H27C	0.9800
C12—C13	1.389 (4)	C28—H28A	0.9800
C12—H12	0.9500	C28—H28B	0.9800
C13—C14	1.378 (4)	C28—H28C	0.9800
C13—H13	0.9500	N3—N3^i^	1.250 (5)
			
C2—C1—C6	120.3 (2)	O2—Si2—C16	110.15 (12)
C2—C1—N1	116.0 (3)	O2—Si2—C15	105.38 (12)
C6—C1—N1	123.7 (2)	C16—Si2—C15	111.68 (16)
C1—C2—C3	121.3 (3)	O2—Si2—C11	108.83 (12)
C1—C2—H2	119.3	C16—Si2—C11	108.79 (13)
C3—C2—H2	119.3	C15—Si2—C11	111.93 (14)
C4—C3—C2	116.9 (2)	Si2—O2—H2O	109.5
C4—C3—Si1	119.7 (2)	Si2—C15—H15A	109.5
C2—C3—Si1	123.3 (2)	Si2—C15—H15B	109.5
C5—C4—C3	122.1 (3)	H15A—C15—H15B	109.5
C5—C4—H4	119.0	Si2—C15—H15C	109.5
C3—C4—H4	119.0	H15A—C15—H15C	109.5
C6—C5—C4	120.2 (3)	H15B—C15—H15C	109.5
C6—C5—H5	119.9	Si2—C16—H16A	109.5
C4—C5—H5	119.9	Si2—C16—H16B	109.5
C5—C6—C1	119.2 (3)	H16A—C16—H16B	109.5
C5—C6—H6	120.4	Si2—C16—H16C	109.5
C1—C6—H6	120.4	H16A—C16—H16C	109.5
O1—Si1—C7	106.33 (15)	H16B—C16—H16C	109.5
O1—Si1—C8	109.67 (14)	C22—C21—C26	120.5 (3)
C7—Si1—C8	112.18 (18)	C22—C21—N3	115.2 (3)
O1—Si1—C3	109.77 (11)	C26—C21—N3	124.3 (3)
C7—Si1—C3	109.72 (14)	C21—C22—C23	121.9 (3)
C8—Si1—C3	109.13 (14)	C21—C22—H22	119.1
Si1—O1—H1O	109.5	C23—C22—H22	119.1
Si1—C7—H7A	109.5	C24—C23—C22	116.6 (3)
Si1—C7—H7B	109.5	C24—C23—Si3	120.7 (2)
H7A—C7—H7B	109.5	C22—C23—Si3	122.7 (2)
Si1—C7—H7C	109.5	C25—C24—C23	122.1 (3)
H7A—C7—H7C	109.5	C25—C24—H24	118.9
H7B—C7—H7C	109.5	C23—C24—H24	118.9
Si1—C8—H8A	109.5	C24—C25—C26	120.1 (3)
Si1—C8—H8B	109.5	C24—C25—H25	119.9
H8A—C8—H8B	109.5	C26—C25—H25	119.9
Si1—C8—H8C	109.5	C21—C26—C25	118.8 (3)
H8A—C8—H8C	109.5	C21—C26—H26	120.6
H8B—C8—H8C	109.5	C25—C26—H26	120.6
N2—N1—C1	112.8 (2)	O3—Si3—C27	109.67 (15)
N1—N2—C9	114.5 (3)	O3—Si3—C28	104.44 (15)
C14—C9—C10	120.2 (2)	C27—Si3—C28	112.7 (2)
C14—C9—N2	125.7 (3)	O3—Si3—C23	109.09 (13)
C10—C9—N2	114.1 (3)	C27—Si3—C23	110.39 (17)
C9—C10—C11	122.0 (3)	C28—Si3—C23	110.32 (17)
C9—C10—H10	119.0	Si3—O3—H3O	109.5
C11—C10—H10	119.0	Si3—C27—H27A	109.5
C12—C11—C10	116.3 (3)	Si3—C27—H27B	109.5
C12—C11—Si2	122.7 (2)	H27A—C27—H27B	109.5
C10—C11—Si2	121.0 (2)	Si3—C27—H27C	109.5
C13—C12—C11	121.8 (3)	H27A—C27—H27C	109.5
C13—C12—H12	119.1	H27B—C27—H27C	109.5
C11—C12—H12	119.1	Si3—C28—H28A	109.5
C14—C13—C12	120.5 (3)	Si3—C28—H28B	109.5
C14—C13—H13	119.7	H28A—C28—H28B	109.5
C12—C13—H13	119.7	Si3—C28—H28C	109.5
C13—C14—C9	119.2 (3)	H28A—C28—H28C	109.5
C13—C14—H14	120.4	H28B—C28—H28C	109.5
C9—C14—H14	120.4	N3^i^—N3—C21	114.0 (3)
			
C6—C1—C2—C3	0.3 (4)	C12—C13—C14—C9	0.3 (4)
N1—C1—C2—C3	179.9 (2)	C10—C9—C14—C13	0.0 (4)
C1—C2—C3—C4	0.3 (4)	N2—C9—C14—C13	178.0 (3)
C1—C2—C3—Si1	−178.2 (2)	C12—C11—Si2—O2	2.3 (3)
C2—C3—C4—C5	−0.4 (4)	C10—C11—Si2—O2	−178.2 (2)
Si1—C3—C4—C5	178.1 (2)	C12—C11—Si2—C16	−117.7 (2)
C3—C4—C5—C6	−0.1 (5)	C10—C11—Si2—C16	61.7 (3)
C4—C5—C6—C1	0.6 (4)	C12—C11—Si2—C15	118.4 (2)
C2—C1—C6—C5	−0.8 (4)	C10—C11—Si2—C15	−62.2 (3)
N1—C1—C6—C5	179.7 (3)	C26—C21—C22—C23	0.7 (5)
C4—C3—Si1—O1	175.9 (2)	N3—C21—C22—C23	179.8 (3)
C2—C3—Si1—O1	−5.7 (3)	C21—C22—C23—C24	−1.1 (4)
C4—C3—Si1—C7	−67.6 (3)	C21—C22—C23—Si3	178.5 (2)
C2—C3—Si1—C7	110.8 (3)	C22—C23—C24—C25	0.4 (5)
C4—C3—Si1—C8	55.7 (3)	Si3—C23—C24—C25	−179.2 (2)
C2—C3—Si1—C8	−125.9 (3)	C23—C24—C25—C26	0.7 (5)
C2—C1—N1—N2	169.9 (2)	C22—C21—C26—C25	0.4 (5)
C6—C1—N1—N2	−10.5 (4)	N3—C21—C26—C25	−178.6 (3)
C1—N1—N2—C9	−178.6 (2)	C24—C25—C26—C21	−1.1 (5)
N1—N2—C9—C14	−0.9 (4)	C24—C23—Si3—O3	45.4 (3)
N1—N2—C9—C10	177.3 (2)	C22—C23—Si3—O3	−134.2 (2)
C14—C9—C10—C11	−0.4 (4)	C24—C23—Si3—C27	166.0 (3)
N2—C9—C10—C11	−178.6 (2)	C22—C23—Si3—C27	−13.6 (3)
C9—C10—C11—C12	0.4 (4)	C24—C23—Si3—C28	−68.8 (3)
C9—C10—C11—Si2	−179.0 (2)	C22—C23—Si3—C28	111.6 (3)
C10—C11—C12—C13	−0.1 (4)	C22—C21—N3—N3^i^	178.2 (3)
Si2—C11—C12—C13	179.4 (2)	C26—C21—N3—N3^i^	−2.7 (5)
C11—C12—C13—C14	−0.3 (4)		

Symmetry code: (i) −*x*+2, −*y*+2, −*z*+2.

### (I) (E)-[Diazene-1,2-diylbis(3,1-phenylene)]bis(dimethylsilanol) Hydrogen-bond geometry (Å, º)

**Table:**

*D*—H···*A*	*D*—H	H···*A*	*D*···*A*	*D*—H···*A*
O1—H1*O*···O2^ii^	0.84	1.87	2.708 (3)	173
O2—H2*O*···O3	0.84	1.86	2.701 (3)	177
O3—H3*O*···O1^iii^	0.84	1.86	2.696 (3)	175

Symmetry codes: (ii) −*x*+1, −*y*+1, −*z*+1; (iii) −*x*+2, −*y*+1, −*z*+1.

### (II) (E)-[Diazene-1,2-diylbis(4,1-phenylene)]bis(dimethylsilanol) Crystal data

**Table:**

C_16_H_22_N_2_O_2_Si_2_	*F*(000) = 1408
*M**_r_* = 330.53	*D*_x_ = 1.206 Mg m^−^^3^
Monoclinic, *P*2_1_/*n*	Mo *K*α radiation, λ = 0.71073 Å
*a* = 17.8705 (4) Å	Cell parameters from 30514 reflections
*b* = 10.0016 (3) Å	θ = 1.6–27.0°
*c* = 20.5323 (5) Å	µ = 0.20 mm^−^^1^
β = 97.013 (2)°	*T* = 200 K
*V* = 3642.36 (16) Å^3^	Block, yellow-orange
*Z* = 8	0.15 × 0.15 × 0.10 mm

### (II) (E)-[Diazene-1,2-diylbis(4,1-phenylene)]bis(dimethylsilanol) Data collection

**Table:**

Stoe IPDS-2 diffractometer	*R*_int_ = 0.026
ω scan	θ_max_ = 27.0°, θ_min_ = 1.6°
30514 measured reflections	*h* = −21→22
7877 independent reflections	*k* = −12→12
6720 reflections with *I* > 2σ(*I*)	*l* = −26→26

### (II) (E)-[Diazene-1,2-diylbis(4,1-phenylene)]bis(dimethylsilanol) Refinement

**Table:**

Refinement on *F*^2^	0 restraints
Least-squares matrix: full	Hydrogen site location: inferred from neighbouring sites
*R*[*F*^2^ > 2σ(*F*^2^)] = 0.037	H-atom parameters constrained
*wR*(*F*^2^) = 0.096	*w* = 1/[σ^2^(*F*_o_^2^) + (0.0466*P*)^2^ + 1.133*P*] where *P* = (*F*_o_^2^ + 2*F*_c_^2^)/3
*S* = 1.04	(Δ/σ)_max_ = 0.001
7877 reflections	Δρ_max_ = 0.32 e Å^−^^3^
409 parameters	Δρ_min_ = −0.20 e Å^−^^3^

### (II) (E)-[Diazene-1,2-diylbis(4,1-phenylene)]bis(dimethylsilanol) Special details

**Table:**

Geometry. All esds (except the esd in the dihedral angle between two l.s. planes) are estimated using the full covariance matrix. The cell esds are taken into account individually in the estimation of esds in distances, angles and torsion angles; correlations between esds in cell parameters are only used when they are defined by crystal symmetry. An approximate (isotropic) treatment of cell esds is used for estimating esds involving l.s. planes.

### (II) (E)-[Diazene-1,2-diylbis(4,1-phenylene)]bis(dimethylsilanol) Fractional atomic coordinates and isotropic or equivalent isotropic displacement parameters (Å^2^)

**Table:**

	*x*	*y*	*z*	*U*_iso_*/*U*_eq_	
C1	0.19059 (8)	0.23669 (15)	0.59401 (8)	0.0364 (3)	
C2	0.22138 (8)	0.17528 (17)	0.54266 (8)	0.0384 (3)	
H2	0.1896	0.1430	0.5055	0.046*	
C3	0.29912 (8)	0.16162 (16)	0.54617 (8)	0.0367 (3)	
H3	0.3200	0.1204	0.5108	0.044*	
C4	0.34777 (8)	0.20714 (14)	0.60064 (8)	0.0327 (3)	
C5	0.31467 (8)	0.26393 (16)	0.65218 (8)	0.0367 (3)	
H5	0.3459	0.2925	0.6904	0.044*	
C6	0.23712 (9)	0.27968 (16)	0.64880 (8)	0.0390 (3)	
H6	0.2159	0.3201	0.6842	0.047*	
Si1	0.45332 (2)	0.20260 (4)	0.60238 (2)	0.03318 (10)	
O1	0.48169 (6)	0.05197 (11)	0.58463 (6)	0.0408 (3)	
H1O	0.4497	−0.0046	0.5934	0.061*	
C7	0.48428 (10)	0.3152 (2)	0.53913 (10)	0.0515 (4)	
H7A	0.5386	0.3043	0.5378	0.077*	
H7B	0.4736	0.4081	0.5500	0.077*	
H7C	0.4570	0.2927	0.4962	0.077*	
C8	0.49909 (9)	0.25256 (18)	0.68462 (9)	0.0461 (4)	
H8A	0.4817	0.1941	0.7180	0.069*	
H8B	0.4859	0.3454	0.6934	0.069*	
H8C	0.5539	0.2446	0.6860	0.069*	
N1	0.11185 (7)	0.26482 (15)	0.59598 (7)	0.0425 (3)	
N2	0.06893 (7)	0.20943 (15)	0.55168 (7)	0.0450 (3)	
C9	−0.00927 (8)	0.24351 (18)	0.55369 (9)	0.0420 (4)	
C10	−0.06127 (9)	0.16317 (19)	0.51704 (10)	0.0486 (4)	
H10	−0.0447	0.0918	0.4918	0.058*	
C11	−0.13769 (9)	0.18688 (18)	0.51714 (9)	0.0445 (4)	
H11	−0.1730	0.1295	0.4927	0.053*	
C12	−0.16403 (8)	0.29287 (16)	0.55217 (8)	0.0363 (3)	
C13	−0.11024 (9)	0.37422 (19)	0.58751 (10)	0.0477 (4)	
H13	−0.1265	0.4477	0.6115	0.057*	
C14	−0.03342 (9)	0.3507 (2)	0.58862 (10)	0.0505 (4)	
H14	0.0022	0.4076	0.6131	0.061*	
Si2	−0.26733 (2)	0.32811 (4)	0.54879 (2)	0.03409 (10)	
O2	−0.31512 (6)	0.19837 (11)	0.51653 (6)	0.0398 (3)	
H2O	−0.3238	0.1456	0.5465	0.060*	
C15	−0.29259 (10)	0.47159 (19)	0.49411 (10)	0.0525 (4)	
H15A	−0.2787	0.4522	0.4504	0.079*	
H15B	−0.2655	0.5513	0.5119	0.079*	
H15C	−0.3470	0.4876	0.4910	0.079*	
C16	−0.29391 (9)	0.3644 (2)	0.63169 (9)	0.0488 (4)	
H16A	−0.3477	0.3865	0.6281	0.073*	
H16B	−0.2642	0.4401	0.6509	0.073*	
H16C	−0.2839	0.2856	0.6598	0.073*	
C21	0.61508 (8)	0.71201 (17)	0.15593 (8)	0.0375 (3)	
C22	0.65679 (9)	0.59473 (17)	0.15840 (9)	0.0415 (4)	
H22	0.6321	0.5105	0.1568	0.050*	
C23	0.73454 (9)	0.60146 (16)	0.16321 (9)	0.0406 (4)	
H23	0.7628	0.5209	0.1645	0.049*	
C24	0.77264 (8)	0.72423 (16)	0.16626 (8)	0.0349 (3)	
C25	0.72921 (9)	0.84003 (16)	0.16432 (9)	0.0414 (4)	
H25	0.7536	0.9246	0.1672	0.050*	
C26	0.65100 (9)	0.83465 (17)	0.15830 (9)	0.0439 (4)	
H26	0.6223	0.9149	0.1558	0.053*	
Si3	0.87781 (2)	0.72742 (4)	0.16980 (2)	0.03419 (10)	
O3	0.90109 (6)	0.66783 (12)	0.10016 (6)	0.0385 (2)	
H3O	0.8680	0.6894	0.0693	0.058*	
C27	0.92173 (10)	0.6144 (2)	0.23463 (9)	0.0525 (5)	
H27A	0.8987	0.5256	0.2290	0.079*	
H27B	0.9139	0.6501	0.2777	0.079*	
H27C	0.9759	0.6075	0.2316	0.079*	
C28	0.91362 (10)	0.90032 (19)	0.18323 (11)	0.0525 (5)	
H28A	0.9685	0.9008	0.1837	0.079*	
H28B	0.9007	0.9336	0.2253	0.079*	
H28C	0.8906	0.9581	0.1477	0.079*	
N3	0.53461 (7)	0.71741 (15)	0.15146 (7)	0.0418 (3)	
N4	0.50481 (7)	0.60550 (15)	0.15605 (8)	0.0444 (3)	
C29	0.42441 (8)	0.61110 (18)	0.15328 (8)	0.0404 (4)	
C30	0.39022 (10)	0.4941 (2)	0.16917 (12)	0.0610 (6)	
H30	0.4195	0.4160	0.1797	0.073*	
C31	0.31279 (10)	0.4912 (2)	0.16968 (13)	0.0619 (6)	
H31	0.2898	0.4106	0.1816	0.074*	
C32	0.26775 (8)	0.60174 (17)	0.15338 (8)	0.0403 (4)	
C33	0.30402 (9)	0.7174 (2)	0.13659 (11)	0.0537 (5)	
H33	0.2749	0.7951	0.1249	0.064*	
C34	0.38159 (10)	0.7227 (2)	0.13647 (11)	0.0548 (5)	
H34	0.4050	0.8030	0.1248	0.066*	
Si4	0.16320 (2)	0.59274 (5)	0.15397 (2)	0.03874 (11)	
O4	0.13140 (6)	0.49686 (12)	0.09145 (6)	0.0399 (3)	
H4O	0.0847	0.4862	0.0908	0.060*	
C35	0.14182 (11)	0.5168 (3)	0.23169 (10)	0.0614 (6)	
H35A	0.0881	0.4944	0.2281	0.092*	
H35B	0.1542	0.5804	0.2677	0.092*	
H35C	0.1718	0.4354	0.2404	0.092*	
C36	0.12030 (11)	0.7614 (2)	0.14383 (12)	0.0616 (5)	
H36A	0.1370	0.8051	0.1054	0.092*	
H36B	0.1360	0.8151	0.1831	0.092*	
H36C	0.0652	0.7532	0.1377	0.092*	

### (II) (E)-[Diazene-1,2-diylbis(4,1-phenylene)]bis(dimethylsilanol) Atomic displacement parameters (Å^2^)

**Table:**

	*U*^11^	*U*^22^	*U*^33^	*U*^12^	*U*^13^	*U*^23^
C1	0.0243 (7)	0.0364 (8)	0.0494 (9)	0.0036 (6)	0.0080 (6)	0.0062 (7)
C2	0.0283 (7)	0.0438 (8)	0.0421 (8)	−0.0007 (6)	0.0008 (6)	0.0016 (7)
C3	0.0287 (7)	0.0406 (8)	0.0416 (8)	0.0022 (6)	0.0071 (6)	0.0003 (6)
C4	0.0244 (6)	0.0324 (7)	0.0417 (8)	0.0024 (5)	0.0054 (6)	0.0041 (6)
C5	0.0283 (7)	0.0393 (8)	0.0426 (8)	0.0007 (6)	0.0053 (6)	−0.0010 (6)
C6	0.0308 (7)	0.0412 (8)	0.0466 (9)	0.0028 (6)	0.0116 (6)	−0.0018 (7)
Si1	0.02102 (18)	0.0348 (2)	0.0440 (2)	0.00213 (15)	0.00485 (16)	0.00273 (17)
O1	0.0256 (5)	0.0385 (6)	0.0595 (7)	0.0017 (4)	0.0104 (5)	−0.0014 (5)
C7	0.0327 (8)	0.0568 (11)	0.0667 (12)	0.0029 (7)	0.0123 (8)	0.0183 (9)
C8	0.0336 (8)	0.0465 (9)	0.0567 (10)	0.0040 (7)	−0.0006 (7)	−0.0046 (8)
N1	0.0282 (6)	0.0496 (8)	0.0498 (8)	0.0009 (6)	0.0058 (6)	0.0017 (6)
N2	0.0306 (7)	0.0527 (8)	0.0516 (8)	0.0044 (6)	0.0052 (6)	−0.0027 (7)
C9	0.0262 (7)	0.0523 (10)	0.0479 (9)	0.0057 (7)	0.0059 (6)	0.0014 (7)
C10	0.0340 (8)	0.0556 (10)	0.0558 (11)	0.0090 (7)	0.0032 (7)	−0.0117 (8)
C11	0.0295 (7)	0.0481 (9)	0.0549 (10)	0.0032 (7)	0.0007 (7)	−0.0087 (8)
C12	0.0257 (7)	0.0412 (8)	0.0418 (8)	0.0020 (6)	0.0033 (6)	0.0004 (6)
C13	0.0295 (8)	0.0516 (10)	0.0613 (11)	0.0014 (7)	0.0035 (7)	−0.0145 (8)
C14	0.0292 (8)	0.0577 (11)	0.0628 (12)	−0.0037 (7)	−0.0012 (8)	−0.0102 (9)
Si2	0.02215 (18)	0.0379 (2)	0.0418 (2)	0.00151 (15)	0.00216 (16)	−0.00057 (17)
O2	0.0321 (5)	0.0449 (6)	0.0413 (6)	−0.0063 (5)	0.0001 (5)	0.0027 (5)
C15	0.0448 (9)	0.0458 (10)	0.0662 (12)	0.0065 (8)	0.0036 (9)	0.0085 (9)
C16	0.0342 (8)	0.0613 (11)	0.0516 (10)	0.0026 (8)	0.0075 (7)	−0.0094 (8)
C21	0.0245 (7)	0.0461 (9)	0.0428 (8)	−0.0018 (6)	0.0083 (6)	−0.0057 (7)
C22	0.0319 (8)	0.0385 (8)	0.0551 (10)	−0.0057 (6)	0.0092 (7)	−0.0067 (7)
C23	0.0309 (7)	0.0357 (8)	0.0558 (10)	0.0014 (6)	0.0077 (7)	−0.0044 (7)
C24	0.0270 (7)	0.0384 (8)	0.0400 (8)	−0.0011 (6)	0.0065 (6)	−0.0029 (6)
C25	0.0289 (7)	0.0359 (8)	0.0604 (10)	−0.0021 (6)	0.0095 (7)	−0.0028 (7)
C26	0.0301 (7)	0.0390 (8)	0.0635 (11)	0.0038 (6)	0.0100 (7)	−0.0042 (8)
Si3	0.02369 (19)	0.0376 (2)	0.0413 (2)	0.00047 (15)	0.00444 (16)	−0.00222 (17)
O3	0.0293 (5)	0.0447 (6)	0.0416 (6)	0.0066 (4)	0.0046 (4)	0.0003 (5)
C27	0.0408 (9)	0.0705 (12)	0.0466 (10)	0.0139 (8)	0.0073 (8)	0.0073 (9)
C28	0.0338 (8)	0.0480 (10)	0.0754 (13)	−0.0065 (7)	0.0055 (8)	−0.0148 (9)
N3	0.0255 (6)	0.0502 (8)	0.0505 (8)	−0.0027 (6)	0.0077 (6)	−0.0058 (6)
N4	0.0266 (6)	0.0522 (8)	0.0550 (9)	−0.0039 (6)	0.0070 (6)	−0.0097 (7)
C29	0.0250 (7)	0.0507 (9)	0.0458 (9)	−0.0047 (6)	0.0056 (6)	−0.0103 (7)
C30	0.0316 (8)	0.0468 (10)	0.1041 (17)	−0.0016 (7)	0.0063 (10)	−0.0007 (10)
C31	0.0309 (8)	0.0484 (10)	0.1067 (18)	−0.0088 (7)	0.0096 (10)	0.0032 (11)
C32	0.0270 (7)	0.0511 (9)	0.0425 (9)	−0.0064 (6)	0.0029 (6)	−0.0097 (7)
C33	0.0293 (8)	0.0553 (11)	0.0764 (13)	0.0002 (7)	0.0054 (8)	0.0086 (9)
C34	0.0320 (8)	0.0536 (11)	0.0792 (14)	−0.0066 (7)	0.0086 (8)	0.0113 (10)
Si4	0.02365 (19)	0.0507 (3)	0.0421 (2)	−0.00499 (17)	0.00473 (16)	−0.00942 (19)
O4	0.0237 (5)	0.0515 (7)	0.0448 (6)	−0.0043 (5)	0.0050 (4)	−0.0091 (5)
C35	0.0390 (9)	0.0998 (17)	0.0458 (10)	−0.0148 (10)	0.0074 (8)	−0.0047 (10)
C36	0.0388 (9)	0.0592 (12)	0.0872 (16)	0.0020 (8)	0.0094 (10)	−0.0177 (11)

### (II) (E)-[Diazene-1,2-diylbis(4,1-phenylene)]bis(dimethylsilanol) Geometric parameters (Å, º)

**Table:**

C1—C6	1.383 (2)	C21—C26	1.383 (2)
C1—C2	1.390 (2)	C21—C22	1.387 (2)
C1—N1	1.4403 (19)	C21—N3	1.4307 (19)
C2—C3	1.389 (2)	C22—C23	1.382 (2)
C2—H2	0.9500	C22—H22	0.9500
C3—C4	1.406 (2)	C23—C24	1.402 (2)
C3—H3	0.9500	C23—H23	0.9500
C4—C5	1.395 (2)	C24—C25	1.392 (2)
C4—Si1	1.8827 (14)	C24—Si3	1.8723 (15)
C5—C6	1.388 (2)	C25—C26	1.389 (2)
C5—H5	0.9500	C25—H25	0.9500
C6—H6	0.9500	C26—H26	0.9500
Si1—O1	1.6446 (12)	Si3—O3	1.6487 (12)
Si1—C8	1.8527 (18)	Si3—C27	1.8466 (19)
Si1—C7	1.8536 (18)	Si3—C28	1.8528 (18)
O1—H1O	0.8400	O3—H3O	0.8400
C7—H7A	0.9800	C27—H27A	0.9800
C7—H7B	0.9800	C27—H27B	0.9800
C7—H7C	0.9800	C27—H27C	0.9800
C8—H8A	0.9800	C28—H28A	0.9800
C8—H8B	0.9800	C28—H28B	0.9800
C8—H8C	0.9800	C28—H28C	0.9800
N1—N2	1.246 (2)	N3—N4	1.248 (2)
N2—C9	1.4439 (19)	N4—C29	1.4321 (19)
C9—C10	1.380 (2)	C29—C34	1.373 (3)
C9—C14	1.388 (3)	C29—C30	1.378 (3)
C10—C11	1.386 (2)	C30—C31	1.385 (2)
C10—H10	0.9500	C30—H30	0.9500
C11—C12	1.395 (2)	C31—C32	1.385 (3)
C11—H11	0.9500	C31—H31	0.9500
C12—C13	1.394 (2)	C32—C33	1.390 (3)
C12—Si2	1.8723 (15)	C32—Si4	1.8719 (15)
C13—C14	1.390 (2)	C33—C34	1.387 (2)
C13—H13	0.9500	C33—H33	0.9500
C14—H14	0.9500	C34—H34	0.9500
Si2—O2	1.6473 (11)	Si4—O4	1.6469 (12)
Si2—C15	1.8438 (19)	Si4—C35	1.849 (2)
Si2—C16	1.8578 (19)	Si4—C36	1.854 (2)
O2—H2O	0.8400	O4—H4O	0.8400
C15—H15A	0.9800	C35—H35A	0.9800
C15—H15B	0.9800	C35—H35B	0.9800
C15—H15C	0.9800	C35—H35C	0.9800
C16—H16A	0.9800	C36—H36A	0.9800
C16—H16B	0.9800	C36—H36B	0.9800
C16—H16C	0.9800	C36—H36C	0.9800
			
C6—C1—C2	119.98 (13)	C26—C21—C22	120.25 (14)
C6—C1—N1	114.13 (14)	C26—C21—N3	115.31 (14)
C2—C1—N1	125.87 (14)	C22—C21—N3	124.44 (14)
C3—C2—C1	119.34 (15)	C23—C22—C21	119.49 (15)
C3—C2—H2	120.3	C23—C22—H22	120.3
C1—C2—H2	120.3	C21—C22—H22	120.3
C2—C3—C4	121.79 (15)	C22—C23—C24	121.62 (15)
C2—C3—H3	119.1	C22—C23—H23	119.2
C4—C3—H3	119.1	C24—C23—H23	119.2
C5—C4—C3	117.20 (13)	C25—C24—C23	117.48 (13)
C5—C4—Si1	120.80 (11)	C25—C24—Si3	122.72 (12)
C3—C4—Si1	121.88 (12)	C23—C24—Si3	119.77 (11)
C6—C5—C4	121.44 (15)	C26—C25—C24	121.45 (15)
C6—C5—H5	119.3	C26—C25—H25	119.3
C4—C5—H5	119.3	C24—C25—H25	119.3
C1—C6—C5	120.19 (15)	C21—C26—C25	119.69 (15)
C1—C6—H6	119.9	C21—C26—H26	120.2
C5—C6—H6	119.9	C25—C26—H26	120.2
O1—Si1—C8	109.59 (7)	O3—Si3—C27	105.89 (8)
O1—Si1—C7	105.95 (8)	O3—Si3—C28	110.33 (8)
C8—Si1—C7	109.78 (9)	C27—Si3—C28	110.81 (10)
O1—Si1—C4	110.68 (6)	O3—Si3—C24	108.57 (6)
C8—Si1—C4	109.91 (8)	C27—Si3—C24	110.72 (8)
C7—Si1—C4	110.86 (7)	C28—Si3—C24	110.40 (8)
Si1—O1—H1O	109.5	Si3—O3—H3O	109.5
Si1—C7—H7A	109.5	Si3—C27—H27A	109.5
Si1—C7—H7B	109.5	Si3—C27—H27B	109.5
H7A—C7—H7B	109.5	H27A—C27—H27B	109.5
Si1—C7—H7C	109.5	Si3—C27—H27C	109.5
H7A—C7—H7C	109.5	H27A—C27—H27C	109.5
H7B—C7—H7C	109.5	H27B—C27—H27C	109.5
Si1—C8—H8A	109.5	Si3—C28—H28A	109.5
Si1—C8—H8B	109.5	Si3—C28—H28B	109.5
H8A—C8—H8B	109.5	H28A—C28—H28B	109.5
Si1—C8—H8C	109.5	Si3—C28—H28C	109.5
H8A—C8—H8C	109.5	H28A—C28—H28C	109.5
H8B—C8—H8C	109.5	H28B—C28—H28C	109.5
N2—N1—C1	114.15 (14)	N4—N3—C21	113.33 (14)
N1—N2—C9	112.70 (14)	N3—N4—C29	113.40 (14)
C10—C9—C14	120.03 (15)	C34—C29—C30	120.02 (15)
C10—C9—N2	115.89 (15)	C34—C29—N4	124.57 (15)
C14—C9—N2	124.07 (15)	C30—C29—N4	115.41 (16)
C9—C10—C11	119.84 (16)	C29—C30—C31	119.46 (18)
C9—C10—H10	120.1	C29—C30—H30	120.3
C11—C10—H10	120.1	C31—C30—H30	120.3
C10—C11—C12	121.69 (16)	C32—C31—C30	122.21 (18)
C10—C11—H11	119.2	C32—C31—H31	118.9
C12—C11—H11	119.2	C30—C31—H31	118.9
C13—C12—C11	117.22 (14)	C31—C32—C33	116.73 (15)
C13—C12—Si2	121.58 (12)	C31—C32—Si4	120.74 (13)
C11—C12—Si2	121.15 (12)	C33—C32—Si4	122.53 (13)
C14—C13—C12	121.79 (16)	C34—C33—C32	121.88 (17)
C14—C13—H13	119.1	C34—C33—H33	119.1
C12—C13—H13	119.1	C32—C33—H33	119.1
C9—C14—C13	119.39 (16)	C29—C34—C33	119.67 (17)
C9—C14—H14	120.3	C29—C34—H34	120.2
C13—C14—H14	120.3	C33—C34—H34	120.2
O2—Si2—C15	106.97 (8)	O4—Si4—C35	110.22 (8)
O2—Si2—C16	110.13 (8)	O4—Si4—C36	110.10 (8)
C15—Si2—C16	109.57 (9)	C35—Si4—C36	110.14 (11)
O2—Si2—C12	109.08 (7)	O4—Si4—C32	105.83 (6)
C15—Si2—C12	109.64 (8)	C35—Si4—C32	109.69 (8)
C16—Si2—C12	111.35 (8)	C36—Si4—C32	110.79 (9)
Si2—O2—H2O	109.5	Si4—O4—H4O	109.5
Si2—C15—H15A	109.5	Si4—C35—H35A	109.5
Si2—C15—H15B	109.5	Si4—C35—H35B	109.5
H15A—C15—H15B	109.5	H35A—C35—H35B	109.5
Si2—C15—H15C	109.5	Si4—C35—H35C	109.5
H15A—C15—H15C	109.5	H35A—C35—H35C	109.5
H15B—C15—H15C	109.5	H35B—C35—H35C	109.5
Si2—C16—H16A	109.5	Si4—C36—H36A	109.5
Si2—C16—H16B	109.5	Si4—C36—H36B	109.5
H16A—C16—H16B	109.5	H36A—C36—H36B	109.5
Si2—C16—H16C	109.5	Si4—C36—H36C	109.5
H16A—C16—H16C	109.5	H36A—C36—H36C	109.5
H16B—C16—H16C	109.5	H36B—C36—H36C	109.5
			
C6—C1—C2—C3	−2.0 (2)	C26—C21—C22—C23	0.0 (3)
N1—C1—C2—C3	176.33 (15)	N3—C21—C22—C23	−179.51 (16)
C1—C2—C3—C4	0.6 (2)	C21—C22—C23—C24	0.6 (3)
C2—C3—C4—C5	1.6 (2)	C22—C23—C24—C25	0.1 (3)
C2—C3—C4—Si1	−174.51 (12)	C22—C23—C24—Si3	−177.88 (14)
C3—C4—C5—C6	−2.5 (2)	C23—C24—C25—C26	−1.2 (3)
Si1—C4—C5—C6	173.69 (12)	Si3—C24—C25—C26	176.63 (14)
C2—C1—C6—C5	1.2 (2)	C22—C21—C26—C25	−1.1 (3)
N1—C1—C6—C5	−177.35 (15)	N3—C21—C26—C25	178.40 (16)
C4—C5—C6—C1	1.1 (2)	C24—C25—C26—C21	1.8 (3)
C5—C4—Si1—O1	131.37 (13)	C25—C24—Si3—O3	−112.31 (15)
C3—C4—Si1—O1	−52.65 (14)	C23—C24—Si3—O3	65.53 (15)
C5—C4—Si1—C8	10.19 (15)	C25—C24—Si3—C27	131.84 (15)
C3—C4—Si1—C8	−173.84 (13)	C23—C24—Si3—C27	−50.33 (16)
C5—C4—Si1—C7	−111.36 (14)	C25—C24—Si3—C28	8.75 (18)
C3—C4—Si1—C7	64.62 (15)	C23—C24—Si3—C28	−173.41 (14)
C6—C1—N1—N2	−170.00 (15)	C26—C21—N3—N4	−173.24 (16)
C2—C1—N1—N2	11.6 (2)	C22—C21—N3—N4	6.3 (2)
C1—N1—N2—C9	−177.93 (14)	C21—N3—N4—C29	178.47 (14)
N1—N2—C9—C10	−165.08 (17)	N3—N4—C29—C34	10.4 (3)
N1—N2—C9—C14	16.0 (3)	N3—N4—C29—C30	−169.46 (18)
C14—C9—C10—C11	−2.4 (3)	C34—C29—C30—C31	−1.7 (3)
N2—C9—C10—C11	178.70 (17)	N4—C29—C30—C31	178.15 (19)
C9—C10—C11—C12	1.7 (3)	C29—C30—C31—C32	1.3 (4)
C10—C11—C12—C13	−0.2 (3)	C30—C31—C32—C33	−0.3 (3)
C10—C11—C12—Si2	177.25 (15)	C30—C31—C32—Si4	179.41 (18)
C11—C12—C13—C14	−0.6 (3)	C31—C32—C33—C34	−0.4 (3)
Si2—C12—C13—C14	−178.11 (16)	Si4—C32—C33—C34	179.90 (16)
C10—C9—C14—C13	1.5 (3)	C30—C29—C34—C33	1.0 (3)
N2—C9—C14—C13	−179.64 (18)	N4—C29—C34—C33	−178.80 (18)
C12—C13—C14—C9	0.0 (3)	C32—C33—C34—C29	0.0 (3)
C13—C12—Si2—O2	−168.44 (14)	C31—C32—Si4—O4	−70.12 (18)
C11—C12—Si2—O2	14.17 (16)	C33—C32—Si4—O4	109.57 (16)
C13—C12—Si2—C15	74.73 (17)	C31—C32—Si4—C35	48.75 (19)
C11—C12—Si2—C15	−102.66 (16)	C33—C32—Si4—C35	−131.55 (17)
C13—C12—Si2—C16	−46.69 (17)	C31—C32—Si4—C36	170.56 (17)
C11—C12—Si2—C16	135.93 (15)	C33—C32—Si4—C36	−9.75 (19)

### (II) (E)-[Diazene-1,2-diylbis(4,1-phenylene)]bis(dimethylsilanol) Hydrogen-bond geometry (Å, º)

**Table:**

*D*—H···*A*	*D*—H	H···*A*	*D*···*A*	*D*—H···*A*
O1—H1*O*···O3^i^	0.84	1.86	2.6686 (15)	161
O2—H2*O*···O4^i^	0.84	1.92	2.7297 (16)	160
O3—H3*O*···O2^ii^	0.84	1.90	2.7010 (15)	160
O4—H4*O*···O1^iii^	0.84	1.87	2.7063 (14)	175

Symmetry codes: (i) *x*−1/2, −*y*+1/2, *z*+1/2; (ii) −*x*+1/2, *y*+1/2, −*z*+1/2; (iii) *x*−1/2, −*y*+1/2, *z*−1/2.

## References

[bb1] Bernstein, J., Davis, E. R., Shimoni, L. & Chang, N.-L. (1995). *Angew. Chem. Int. Ed. Engl.* **34**, 1555–1573.

[bb2] Brandenburg, K. (1999). *DIAMOND*. Crystal Impact GbR, Bonn, Germany.

[bb3] Etter, M. C., MacDonald, J. C. & Bernstein, J. (1990). *Acta Cryst.* B**46**, 256–262. 10.1107/s0108768189012929 2344397

[bb4] Groom, C. R., Bruno, I. J., Lightfoot, M. P. & Ward, S. C. (2016). *Acta Cryst.* B**72**, 171–179. 10.1107/S2052520616003954 PMC4822653 27048719

[bb5] Kano, N., Komatsu, F. & Kawashima, T. (2001). *Chem. Lett.* **30**, 338–339.

[bb6] Kano, N., Yamamura, M., Komatsu, F. & Kawashima, T. (2003). *J. Organomet. Chem.* **686**, 192–197.

[bb7] Kizilkan, E., Strüben, J., Jin, X., Schaber, C. F., Adelung, R., Staubitz, A. & Gorb, S. N. (2016). *R. Soc. Open Sci.* **3**, 150700. 10.1098/rsos.150700 PMC4852635 27152212

[bb8] Lagrasta, C., Bellobono, I. R. & Bonardi, M. (1997). *J. Photochem. Photobiol. A*, **110**, 201–205.

[bb9] Sheldrick, G. M. (2008). *Acta Cryst.* A**64**, 112–122. 10.1107/S0108767307043930 18156677

[bb10] Sheldrick, G. M. (2015). *Acta Cryst.* C**71**, 3–8.

[bb11] Stoe (2008). *X-AREA*, *X-RED32* and *X-SHAPE*. Stoe & Cie, Darmstadt, Germany.

[bb12] Strüben, J., Gates, P. J. & Staubitz, A. (2014). *J. Org. Chem.* **79**, 1719–1728. 10.1021/jo402598u 24502513

[bb13] Strüben, J., Lipfert, M., Springer, J.-O., Gould, C. A., Gates, P. J., Soennichsen, F. D. & Staubitz, A. (2015). *Chem. Eur. J.* **21**, 11165–11173. 10.1002/chem.201500003 26118826

[bb14] Westrip, S. P. (2010). *J. Appl. Cryst.* **43**, 920–925.

[bb15] Yamamura, M., Kano, N. & Kawashima, T. (2009). *Z. Anorg. Allg. Chem.* **635**, 1295–1299.

[bb16] Yesodha, S. K., Sadashiva, C. K., Pillai, P. & Tsutsumi, N. (2004). *Prog. Polym. Sci.* **29**, 45–74.

[bb17] Yu, Y., Nakano, M. & Ikeda, T. (2003). *Nature*, **425**, 145. 10.1038/425145a 12968169

